# TPD7 inhibits the growth of cutaneous T cell lymphoma H9 cell through regulating IL‐2R signalling pathway

**DOI:** 10.1111/jcmm.14810

**Published:** 2019-11-19

**Authors:** Man Zhu, Liu Yang, Xianpeng Shi, Zhengyan Gong, Runze Yu, Dongdong Zhang, Yanmin Zhang, Weina Ma

**Affiliations:** ^1^ School of Pharmacy Health Science Center Xi'an Jiaotong University Xi'an China; ^2^ State Key Laboratory of Shaanxi for Natural Medicines Research and Engineering Xi’an China

**Keywords:** cell growth, cutaneous T cell lymphoma, H9, IL‐2R, TPD7

## Abstract

IL‐2R pathway is a key regulator in the development of immune cells and has emerged as a promising drug target in cancer treatment, but there is a scarcity of related inhibitors. TPD7 is a novel biphenyl urea taspine derivate, which has been shown anti‐cancer effect. Here, we demonstrated the anti‐cancer activity of TPD7 in cutaneous T cell lymphoma and investigated the underlying mechanism of TPD7 through IL‐2R signalling. The inhibitory effect of TPD7 on cell viability exhibited a strong correlation with the expression level of IL‐2R, and cutaneous T cell lymphoma H9 and HUT78 cells were most sensitive to TPD7. TPD7 was nicely bound to IL‐2R and down‐regulated the mRNA and protein levels of IL‐2R. Furthermore, TPD7 suppressed the downstream cascades of IL‐2R including JAK/STAT, PI3K/AKT/mTOR and PLCγ/Raf/MAPK signalling, resulting in Bcl‐2 mitochondrial apoptosis pathway and cell cycle proteins CDK/Cyclins regulation. And, these were verified by flow cytometry analysis that TPD7 facilitated cell apoptosis in H9 cells via mitochondrial pathway and impeded cell cycle progression at G2/M phase. TPD7 is a novel anti‐cancer agent and may be a potential candidate for cutaneous T cell lymphoma treatment by regulating IL‐2R signalling pathway.

## 1 INTRODUCTION

Cutaneous T cell lymphoma (CTCL) is a kind of extranodal non‐Hodgkin T cell lymphoma characterized by the presence of malignant T cells in the skin. Among various forms of CTCLs, mycosis fungoides (MF) and the leukaemic variant Sézary syndrome (SS) are the most prevalent forms of CTCL.^1^, ^2^ Although some induction chemotherapies have been approved for the treatment of CTCL, the responses of many patients are typically short‐lived.^3^ Therefore, developing effective chemotherapies for CTCL is extremely urgent.

Interleukin‐2 (IL‐2), a 15.5 kD type 1 four α‐helical bundle cytokine, was first discovered in 1976s as a T cell growth factor present in supernatants of activated human T cells.^4^, ^5^ It is well‐documented that IL‐2 exerts its biological activities through binding to three classes of cell surface IL‐2 receptors (IL‐2R), IL‐2Rα (CD25), IL‐2Rβ (CD122) and IL‐2Rγ (CD132).^6^, ^7^ Interestingly, IL‐2Rα subunit has no effect on signal transduction but primarily increases the affinity of ligand binding, while the β and γ subunits not only participate in ligand binding, but also exert crucial functions during the IL‐2/IL‐2R signal transduction through associated with the Janus kinases (JAK).^8^ IL‐2 binds with low affinity to IL‐2Rα, while the βγ dimer binding IL‐2 shows intermediate affinity and the highest affinity binding form is the αβγ trimeric complex.^9^

Following receptor binding, IL‐2 activates three principal signalling pathways: phosphatidylinositol 3‐kinase (PI3K)‐AKT, Ras‐mitogen‐activated protein kinase (MAPK), and Janus kinase (JAK)‐signal transducer and activator of transcription (STAT) pathways, which mediate cell growth, survival, activation‐induced cell death (AICD) and differentiation.^10^ The IL‐2 signal transduction mainly occurs via activation of JAK1 and JAK3 (associated with IL‐2Rβ and IL‐2Rγ, respectively). The key tyrosine residues present in the cytoplasmic domain of the IL‐2R are activated following phosphorylation of JAKs, which subsequently serve as a docking site and recruit the adaptor protein SHC and STAT molecules (including STAT1, STAT3 and STAT5).^11^ After tyrosine‐phosphorylated by JAKs, STATs are released from IL‐2R and then moved to the nucleus through dimerization to modulate the expression of downstream target genes.^12^ Besides, SHC serves just like a platform to activate Raf1 and thereafter activates MAPK and eventually phosphorylates ERK, which participates in cell mitosis.^13^, ^14^ Additionally, IL‐2Rβ triggering activates the molecular AKT/PKB with the upstream PI3K and then induces mTOR activation whose major role is closely associated with cell growth, differentiation and apoptosis.^13^, ^14^ These suggest that IL‐2 signal transduction is indeed intricate and criss‐crossed to exert its immune regulation. Nevertheless, tumour immune escape and malignant growth will happen if aberrant responses of IL‐2 occurs. It has been reported that, in malignant CD4^+^ T cells derived from the CTCL, both of activated PI3K/AKT and MEK/ERK pathways contribute to mTORC1 activation, which appears to depend on the IL‐2‐induced γc/Jak signalling.^15^ IL‐2R has proven to be an attractive target for immune intervention and T cell‐directed therapies.^16^ To date, the IL‐2R target agents, such as daclizumab, basiliximab and denileukin diftitox, have demonstrated activity in diseases characterized by proliferation of activated T cells, but most of them are mainly used to protect against the rejection of certain organ transplants or cannot block IL‐2 signalling activated by intermediate affinity binding.^17^, ^18^, ^19^ Importantly, daclizumab and basiliximab are two monoclonal antibodies to human IL‐2Rα and there is no related small molecule compound targeting IL‐2R on the market. Consequently, there is an urgent and unmet need to develop effective chemotherapies that can target IL‐2R and block downstream signalling.

**Figure 1:**
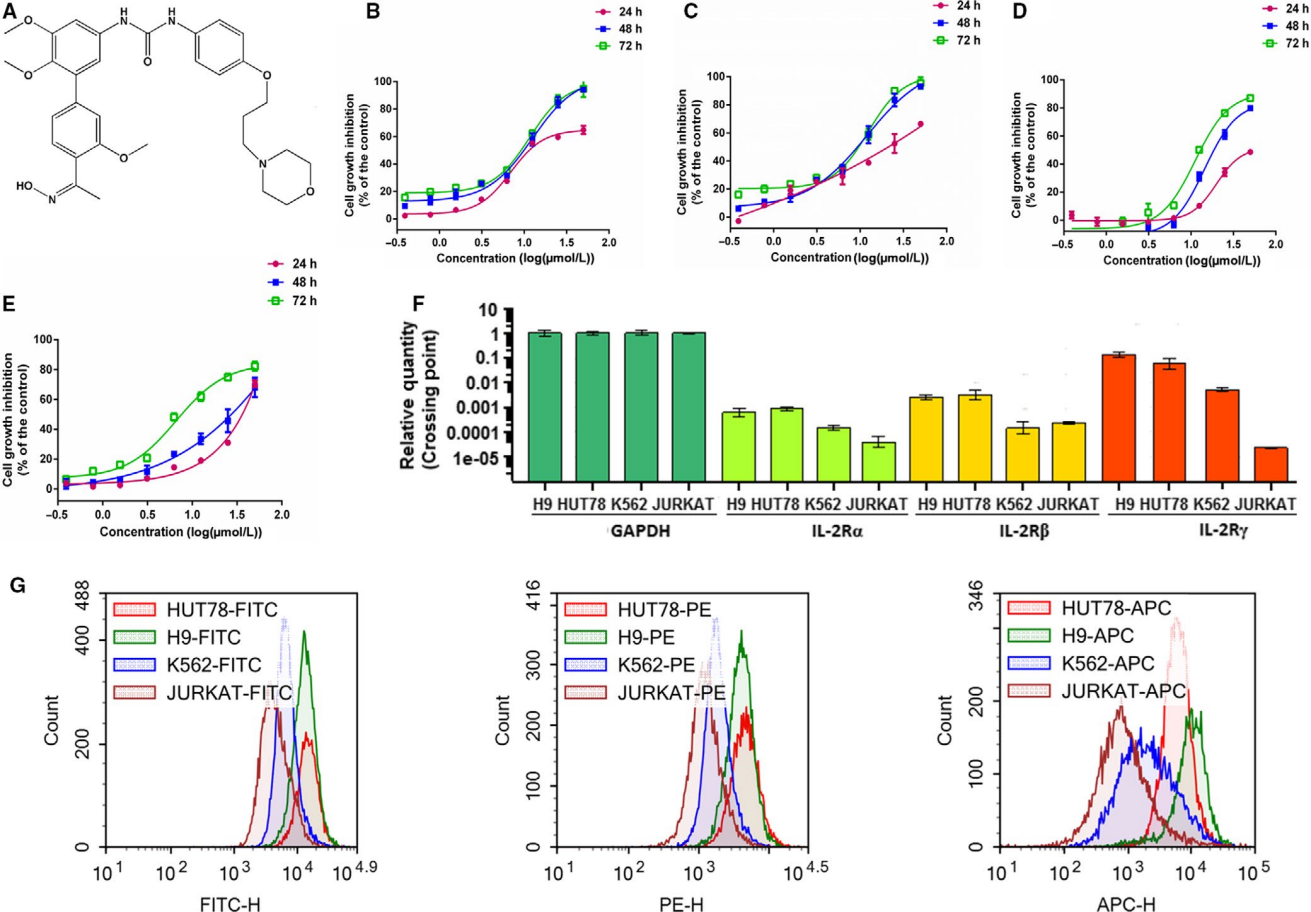
The effect of TPD7 on leukaemia cell lines proliferation. A, chemical structure of TPD7; B‐E, effects of TPD7 on cell proliferation in H9, HUT78, JURKAT and K562 cells were determined by MTT assay, respectively. Cells were treated with TPD7 (0.39‐50 μmol/L) for 24, 48 and 72 h, respectively; F, The transcriptional level of IL‐2Rα, IL‐2Rβ and IL‐2Rγ in haematologic cancer cells; G, cells were stained with IL‐2Rα FITC, IL‐2Rβ PE and IL‐2Rγ APC and determined by flow cytometry. Data are presented as the mean ± standard deviation obtained from three independent experiments

TPD7 (N‐[40‐Acetyl‐30,5,6‐trimethoxybiphenyl‐3‐yl]‐N0‐[4‐[3‐morpholin‐4‐ ylpropoxy]phenyl]urea; Figure 1A), a novel biphenyl urea taspine derivative, was designed and synthesized in our laboratory.^20^ Studies have shown that through targeting VEGFR2, TPD7 could inhibit cancer cell growth effectively.^20^ In this study, we sought to investigate whether TPD7 could modulate IL‐2R signalling and thus inhibit cutaneous T cell lymphoma growth.

## 2 MATERIALS AND METHODS

### 2.1 Chemicals and reagents

TPD7 was from the Research and Engineering Center for Natural Medicine, Xi'an Jiaotong University (Xi'an, China). The human haematologic cancer cell lines (H9, HUT78, JURKAT and K562) were obtained from Shanghai Institute of Cell Biology in the Chinese Academy of Sciences (Shanghai, China). RPMI 1640 medium, 3‐(4,5‐Dimethylthiazol‐2‐yl)‐2.5‐diphenyl‐2H‐tetrazolium bromide (MTT), Iscove's modified Dulbecco's medium (IMDM), was purchased from Sigma‐Aldrich and foetal bovine serum (FBS) was obtained from ExCell Bio Co., Ltd. Protease and phosphatase inhibitors were purchased from Roche Tech. RIPA Lysis Buffer, BCA protein assay reagent kit, dimethylsulphoxide (DMSO) and the RNAfast200 kit were from Pioneer Biotechnology Co., Ltd. Enhanced Chemiluminescent (ECL) Plus Reagent kit was obtained from 4A Biotech Co., Ltd. PrimeScript RT Master Mix Perfect Real Time kit, SYBR^®^ Premix Ex TaqTM II and a Thermal Cycle Dice Real time system were from TaKaRa biotechnology. FITC anti‐human CD25 (IL‐2Rα) antibody, PE anti‐human CD122 (IL‐2Rβ) antibody and APC anti‐human CD132 (common γ chain) antibody were obtained from BioLegend. Annexin V‐FITC apoptosis detection kit was purchased from Pioneer Biotechnology Co., Ltd. RNase and propidium iodide were from Sigma‐Aldrich. IL‐2Rα rabbit mAb, PI3K p110α rabbit mAb, PI3K p110β rabbit mAb, PI3K p110γ rabbit mAb, PI3K p85 rabbit mAb, p44/42 MAPK(ERK1/2) rabbit mAb, phospho‐p44/42 MAPK(p‐ERK1/2) rabbit mAb, phospho‐MEK1/2 rabbit mAb, MEK1/2 rabbit mAb, phospho‐PLC‐γ rabbit mAb, PLC‐γ rabbit mAb, phospho‐mTOR rabbit mAb, mTOR rabbit mAb, phospho‐AKT rabbit mAb, AKT rabbit mAb, phospho‐p38 (Thr180/Tyr182) rabbit mAb, p38 rabbit mAb, CDC2 rabbit mAb, Bak rabbit mAb, GAPDH rabbit mAb and goat anti‐rabbit IgG were purchased from cell signalling. IL‐2Rβ rabbit mAb, IL‐2Rγ rabbit mAb, cyclinB1 rabbit mAb, cyclinD1 rabbit mAb, cyclinE rabbit mAb, CDK2 rabbit mAb, cyclinA2 rabbit mAb, Bad rabbit mAb, Bax rabbit mAb, Mcl‐1 rabbit mAb, Bcl‐2 rabbit mAb, p53 rabbit mAb and Raf rabbit mAb were obtained from Protein technology Group.

### 2.2 Cell culture

H9 and JURKAT cells were cultured in RPMI 1640 medium supplemented with 10% heat‐inactivated foetal bovine serum (FBS), 100 U/mL penicillin and 100 U/mL streptomycin at 37°C, 5% CO_2_ and 95% humidity. HUT78 and K562 cells were cultured in IMDM medium supplemented with 10% heat‐inactivated FBS, 100 U/mL penicillin and 100 U/mL streptomycin at 37°C, 5% CO_2_ and 95% humidity.

### 2.3 Cell viability assay

3‐(4,5‐Dimethylthiazol‐2‐yl)‐2.5‐diphenyl‐2H‐tetrazolium bromide assay was used to determine the inhibitory effect of TPD7 on cell viability. Growing cells were incubated at a density of 2 × 10^4^ cells per well in a 96‐well plate overnight and then treated with the indicated concentrations of TPD7 (0.39 to 50 μmol/L) for 24, 48 and 72 hours, respectively. Then, MTT solution (5 mg/mL and 10 μL) was added to each well and incubated for an additional 4 hours. Precipitated formazan was solubilized with 150 µL of DMSO, and the absorbance was measured by the micro‐plate reader at a wavelength of 490 nm.

### 2.4 Quantitative real‐time PCR assay

Total RNA was isolated using the RNAfast 200 kit following the manufacturer's protocol and was converted into cDNA using the PrimeScript RT Master Mix Perfect Real Time kit. Real‐time PCR was performed by SYBR^®^ Premix Ex TaqTM II and a Thermal Cycle Dice Real time system. In a final volume of 25 µL in a capillary tube, 2 µL of cDNA samples, 12.5 µL SYBR^®^ Premix Ex TaqTM II, 0.5 µL of each forward and reverse primers (20 μmol/L) and 8.5 µL of nuclease‐free water were added to conduct real‐time PCR. The results were analysed using the manufacturer's program (Thermal Cycler Dice^TM^ Real Time System). The relative amount of mRNA for each gene was normalized based on that of the housekeeping gene GAPDH. The primer sequences were as following:

GAPDH forward primer: 5ʹ‐AAGGCTGTGGGCAAGGTCATC‐3ʹ;

GAPDH reverse primer:5ʹ‐GCGTCAAAGGTGGAGGAGTGG‐3ʹ;

IL‐2Rα forward primer: 5ʹ‐CTTCCTGCCTCGTCACAAC‐3ʹ;

IL‐2Rα reverse primer: 5ʹ‐TCTTCTACTCTTCCTCTGTCTCC‐3ʹ;

IL‐2Rβ forward primer: 5ʹ‐TGAGAACCTTCGCCTGATG‐3ʹ;

IL‐2Rβ reverse primer: 5ʹ‐CATTCCTGCTTCTGCTTGAG‐3ʹ;

IL‐2γ forward primer: 5ʹ‐ATGGGCAGAAACGCTACAC‐3ʹ;

IL‐2γ reverse primer: 5ʹ‐CAGAGATAACCACGGCTTCC‐3ʹ.

### 2.5 Cell surface flow cytometry staining

Cell surface flow cytometry staining was performed according to the manufacturer's protocol. All stained cells were analysed by NovoCyte 2040R Flow Cytometer (ACEA).

### 2.6 Microarray analysis

H9 cells were plated into 6‐well plates one day prior to treatment with TPD7 (0 or 3.12 μmol/L) for 48 hours. Total RNA was isolated using Trizol. Microarray analysis and data processing were done by Gminix.

### 2.7 Molecular docking study

Protein‐ligand docking is widely used to predict the structure and the binding mode of certain ligand with protein. Herein, a docking study was performed using SYBYL‐X 1.1 to understand the molecular binding mode of TPD7 with IL‐2R domain (PDB ID: 2ERJ). The Sybyl/Sketch module was used to construct the substrate, and Powell's method was used to optimize the substrate. Tripos force field and Gasteiger‐Hückel charges were used to minimize energy with the convergence criterion set at 0.005 kcal/(Å mol) and the maximum set at 1000 iterations. A non‐bonded cut‐off distance was set at 8 Å to explore the intramolecular interaction.

### 2.8 Flow cytometric analysis of cell cycle distribution

To analyse the distribution of cell cycle stage, growing H9 or HUT78 cells in 6‐well plate were incubated with TPD7 at indicated concentration for 48 hours and then the cells were harvested by centrifugation. The cells were washed with cold PBS and then fixed in cold 70% ethanol to store at 4°C overnight. The fixed cells were centrifuged, washed twice with ice‐cold PBS and incubated with RNase A (50 μg/mL) for 30 minutes, followed by incubation with PI (60 μg/mL) away from light for another 30 minutes. Stained H9 cells were analysed using a BD FACSCalibur Flow cytometer, and stained HUT78 cells were analysed by NovoCyte 2040R Flow Cytometer.

### 2.9 Flow cytometric analysis of apoptosis

After exposed to different concentrations of TPD7 (0, 1.56, 3.12 and 6.25 μmol/L) for 48 hours, H9 cells were harvested and washed twice with PBS. Cell apoptosis analysis was conducted by Annexin V‐FITC Apoptosis Kit following to the manufacturer's instructions. All stained cells were analysed by BD FACSCalibur Flow cytometer. Cells with Annexin V‐FITC and PI double positive signals were combined for apoptosis induction analysis.

### 2.10 Determination of mitochondrial transmembrane potential (Δψm)

H9 cells were seeded in 6‐well plate at a proper density. After 24 hours incubation, cells were treated with TPD7 at concentrations of 0, 1.56, 3.12 or 6.25 μmol/L for 48 hours and then were washed with RPMI 1640 medium, followed by 30 minutes incubation with 1 mmol/L Rhodamine 123 at 37°C away from light. Cells were washed with RPMI 1640 medium again, and the fluorescent intensity was measured using a BD FACSCalibur Flow cytometer.

### 2.11 Western blot analysis

H9 or HUT78 cells in 6‐well plates were treated with TPD7 at indicated concentration and then incubated for 48 hours. Total cellular proteins were extracted using a RIPA buffer containing protease inhibitor cocktail. After quantification using a BCA kit, equivalent amounts of proteins were loaded to a 12% SDS‐polyacrylamide gel electrophoresis and transferred onto polyvinylidene difluoride membranes. Membranes were thereafter blocked with 5% non‐fat milk in Tris‐buffered saline containing 0.05% Tween 20 (TBST) buffer at room temperature for 2 hours and then incubated with individual primary monoclonal antibodies in TBST buffer at 4°C, followed by incubation with corresponding secondary antibodies in TBST buffer at 37°C for 1 hours. The immunoreactive proteins on the membranes were detected using an enhanced chemiluminescence (ECL) kit. Band intensity was quantified by densitometric analyses using the Image‐Pro Plus software, which was provided by Media Cybernetics Inc.

### 2.12 Statistical analysis

Data were analysed by GraphPad Prism built‐in tests. All values are presented as the mean ± standard deviation obtained from three independent experiments. Number of samples analysed per experiment and whether data are the mean of multiple experiments, or selected as representative, are indicated in Figure legends. Student's *t* test was used to compare individual data with control values. All statistical tests were two‐sided. Statistical analysis was performed using the statistical software SPSS18.0 and ANOVA was used to analyse statistical differences between groups under different conditions. Differences were considered statistically significant at a *P* value <.05. *P < .05, **P < .01 and ***P < .001 compared with control group. Data are presented as mean ± SEM

## 3 RESULTS

### 3.1 The sensitivity of cancer cells to TPD7 is positively related to the IL‐2R expression

In order to assess the effects of TPD7 on haematologic cancer cells, cutaneous T cell lymphoma H9 cells and HUT78 cells, acute T cell lymphoma JURKAT cells and chronic myeloid leukaemia K562 cells were treated with TPD7 at concentrations of 0.39, 0.78, 1.56, 3.12, 6.25, 12.50, 25.00 and 50.00 μmol/L for 24, 48 and 72 hours, respectively. The results showed that H9 cells were much more sensitive to TPD7 compared with HUT78, K562 and JURKAT cells (Figure 1B‐E). The IC_50_ values of H9, HUT78, K562 and JURKAT cells with TPD7 treatment for 48 hours were 11.56, 11.95, 20.20 and 26.44 μmol/L, respectively. We next addressed the mechanistic basis of TPD7‐induced potent inhibitory effect on H9 cells, which were the most sensitive to TPD7. Notably, it has been documented that T cell growth factor (TCGF, also known as IL‐2) was specifically produced in cutaneous T cell lymphoma H9 cells.^21^ We speculated that the inhibitory effect of TPD7 on H9 cells may be partially due to IL‐2R pathway. Surprisingly, we found that H9 cells had higher expression of IL‐2Rs than the other three cell lines at mRNA level (Figure 1F). And, flow cytometry results also demonstrated that FITC‐/PE‐/APC‐labled H9 cells had stronger fluorescence intensity than other three labelled cells (Figure 1G), indicating that the level of IL‐2Rs at the H9 cell surface was higher than that of other three cell lines.

### 3.2 The interaction of TPD7 and IL‐2R

**Figure 2:**
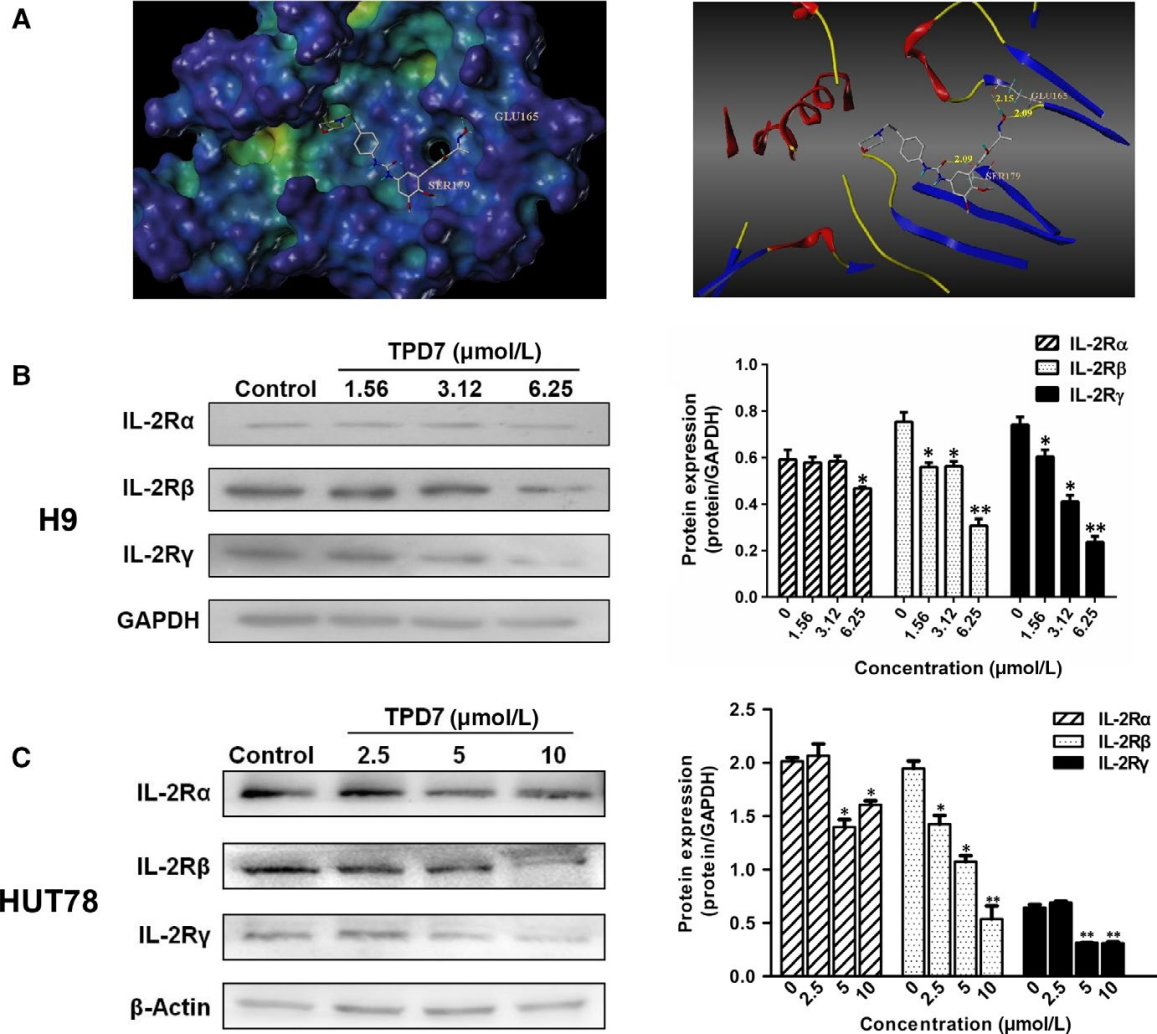
The interaction between TPD7 and IL‐2R. A, docked molecule (TPD7) in the crystal structure of IL‐2R (PDB ID: 2ERJ). Hydrogen bonds were depicted in dashed yellow lines; B, levels of IL‐2Rα, IL‐2Rβ and IL‐2Rγ in H9 cells treated with TPD7 (1.56, 3.12, or 6.25 μmol/L) for 48 h were examined by Western blot assay; C, levels of IL‐2Rα, IL‐2Rβ and IL‐2Rγ in HUT78 cells treated with TPD7 (2.5, 5, or 10 μmol/L) for 48 h were examined by Western blot assay. Data are presented as the mean ± standard deviation obtained from three independent experiments. *P < .05, **P < .01 and ***P < .001 vs. the control group

To further verify the speculation, the affinity of TPD7 bound to the active site of IL‐2R was evaluated using molecular docking studies. The binding mode of TPD7 with IL‐2R was shown in Figure 2A. TPD7 occupied in the ATP‐pocket of IL‐2R with three hydrogen bonds listed as follows. N‐(pyridin‐2‐yl)acrylamide in TPD7 formed one hydrogen bond with Ser 179 in the hinge region of IL‐2R with distance of 2.09 Å, and 1H‐indazol‐3‐amine formed two hydrogen bonds with Glu 165 in the hinge region of IL‐2R with the distance of 2.09 Å and 2.15 Å, respectively. The docking results demonstrated that TPD7 fit well with IL‐2R.

Meanwhile, the effect of TPD7 on the protein level of IL‐2Rs was detected by Western blot. Both of IL‐2Rβ and IL‐2Rγ were decreased at protein level in TPD7‐treated H9 and HUT78 cells (Figure 2B, 2), which suggested that TPD7 could inhibit CTCL cell growth through regulating the level of IL‐2Rs.

### 3.3 TPD7 induced IL‐2R‐related differentially expressed genes

**Figure 3:**
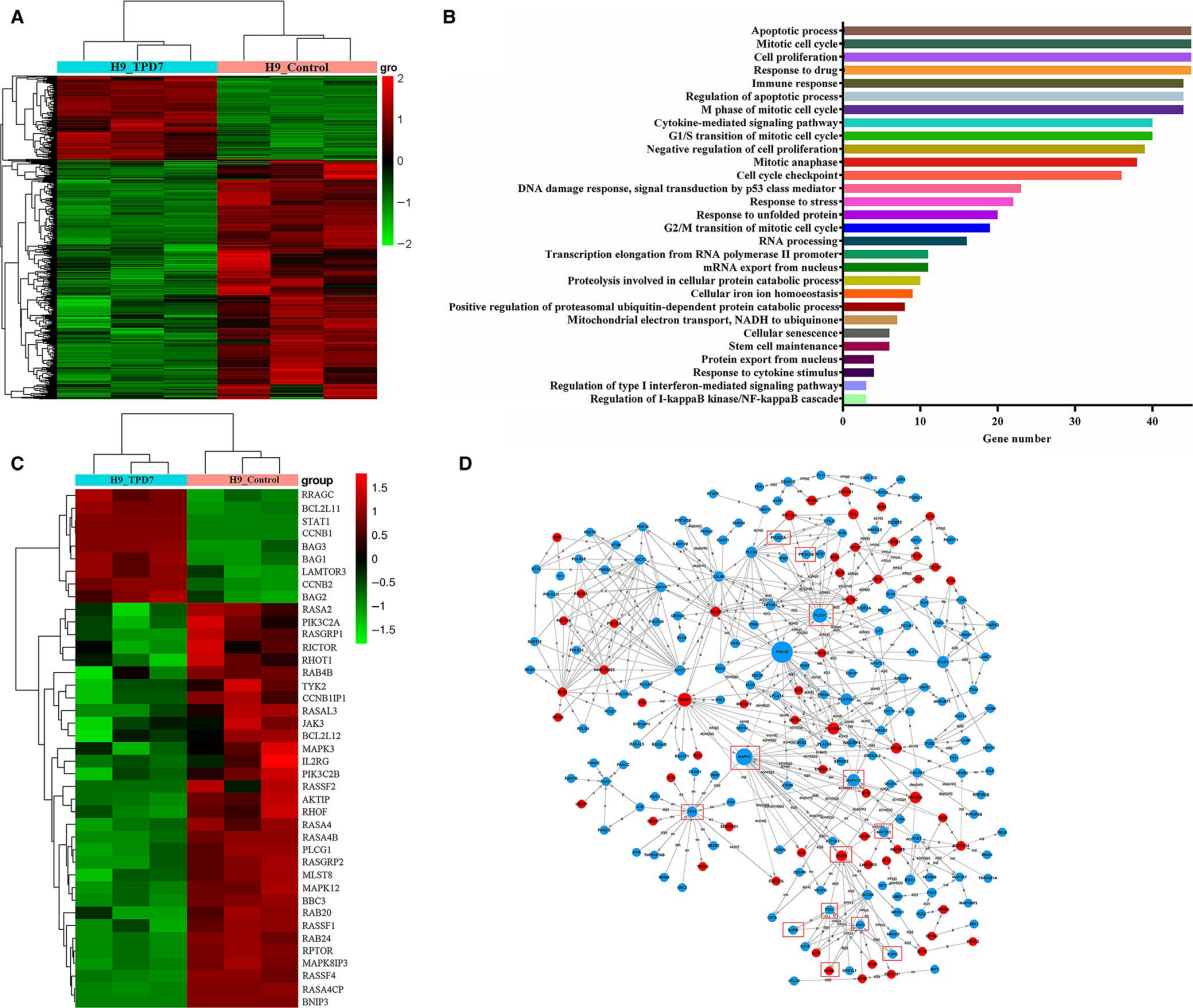
The differentially expressed genes induced by TPD7. A, heatmap shows clustering by gene expression in H9 cells and TPD7‐treated H9 cells; B, Gene Ontology terms with the differential expression between H9 cells and TPD7‐treated H9 cells; C, the IL‐2R related differential gene expression of untreated control and H9 cells exposed to TPD7; D, TPD7‐regulated gene network analysis. The concentration of TPD7 is 3.12 μmol/L. Each column represents one sample, and each gene was depicted by one row, where red denotes an increase in gene expression and green denotes a decrease in gene expression as compared with the other group. Data are presented as the mean ± standard deviation obtained from three independent experiments

Given these findings, we are highly intrigued by the question of whether the interaction of TPD7 and IL‐2R could contribute to the differential gene expression in TPD7‐induced inhibition of cell proliferation. We investigated the differential gene expression of H9 cells exposed to TPD7. Unsupervised hierarchical clustering showed that wild‐type H9 cells and TPD7‐treated H9 cells had distinct patterns of gene expression (Figure 3A). The expression profiles of the two groups of cells differed with respect to two major groups of GSEA‐GO terms (Figure 3B). Firstly, TPD7 treatment showed significantly different expression of genes involved in cell apoptotic process. Secondly, TPD7 treatment regulated genes involved in cell division and the cell cycle machinery when compared with H9 cells. Importantly, consistent with the protein level differences, both of IL‐2Rβ and IL‐2Rγ genes were significantly down‐regulated in TPD7‐treated H9 cells (Figure 3C, 3). And, the major molecules of JAK/STAT, PI3K/AKT and MAPK pathway, the validated IL‐2R substrates, were significantly decreased by TPD7 (Figure 3C, 3). Hence, we conclude that TPD7 treatment leads to down‐regulation of IL‐2R signalling, supporting its key role against CTCL H9 cells.

### 3.4 TPD7 induces G2/M phase arrest in H9 and HUT78 cells

**Figure 4:**
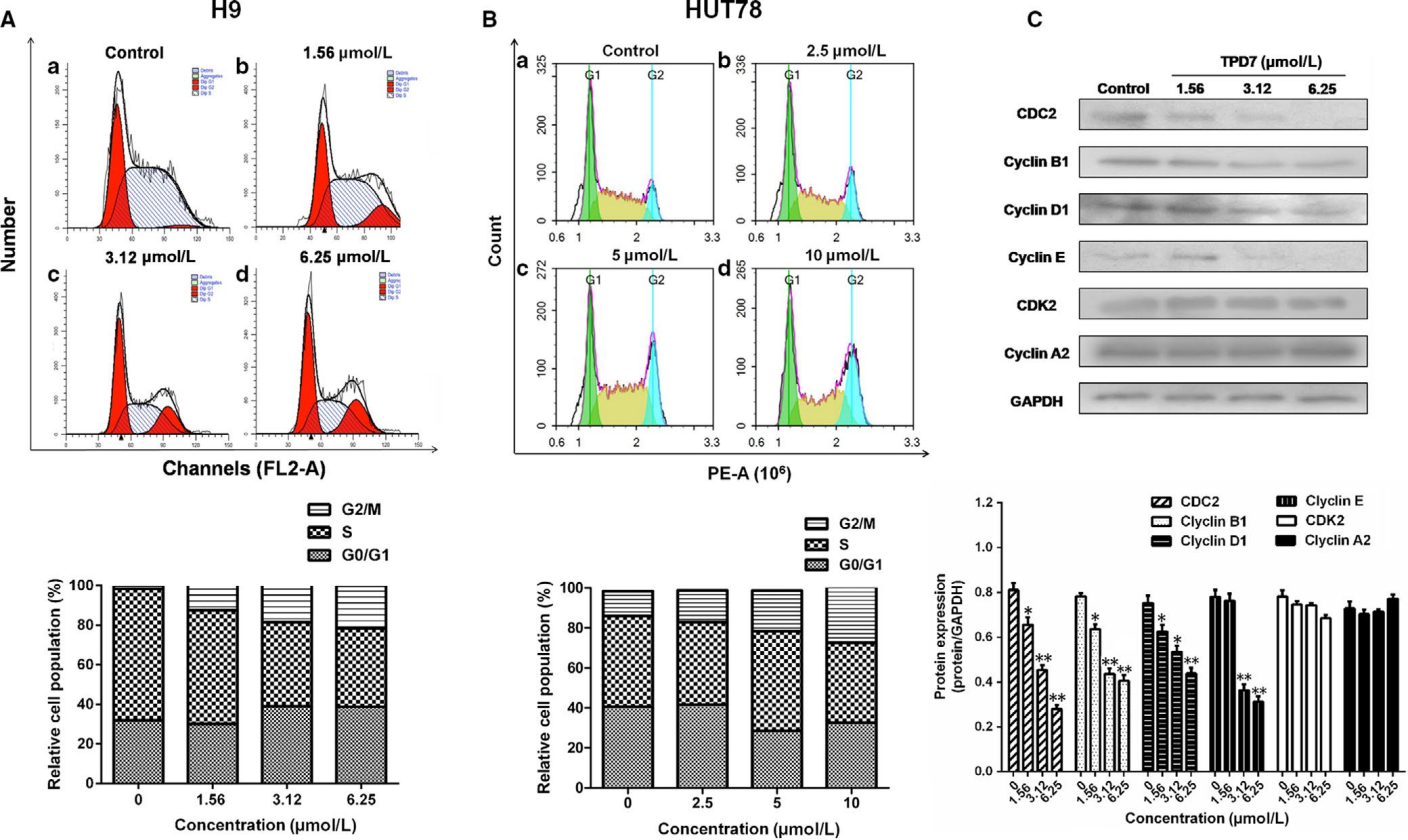
Effects of TPD7 treatment on cell cycle distribution. A, representative flow cytometric DNA content histogram of untreated control and H9 cells exposed to 1.56, 3.12 and 6.25 μmol/L TPD7, respectively; B, representative flow cytometric DNA content histogram of untreated control and HUT78 cells exposed to 2.5, 5 and 10 μmol/L TPD7, respectively; C, effects of TPD7 on cell cycle‐related protein level. Data are presented as the mean ± standard deviation obtained from three independent experiments. *P < .05, **P < .01 and ***P < .001 vs. the control group

To gain insight into the mechanisms responsible for TPD7‐mediated H9 cell growth inhibition, we also assessed the effect of TPD7 on cell cycle arrest. As shown in Figure 4A, TPD7 treatment produced a remarkable increase in the percentage of cells in the G2/M phase with a parallel significant reduction in cells in the S phase in H9 cells, compared to the control. As the concentration of TPD7 increased from 1.56, 3.16‐6.25 μmol/L, the population of H9 cells in G2/M phase increased to 12.44%, 18.54% and 21.60% compared with the untreated control (1.56%), which showed a dose‐dependent manner. Meanwhile, TPD7 was able to increase the percentage of H9 cells in G1 phase at a moderate extent. The results suggested a potential blockade of cell cycle progression induced by TPD7 at the G2/M checkpoint in H9 cells. Moreover, blockade of cell cycle progression at G2/M phase were also observed in TPD7‐treated HUT78 cells (Figure 4B).

To examine whether the change of cell cycle regulatory proteins confers cell cycle arrest in H9 cells, Western blot analysis was performed to examine the levels of CDK/Cyclins proteins. Figure 4C showed that the amount of CDC2, cyclinB1, cyclinD1 and cyclinE was gradually decreased after treatment of TPD7 at different concentrations, whereas the protein level of CDK2 and cyclinA2 remained unchanged in TPD7‐treated H9 cells.

### 3.5 TPD7 induces cell apoptosis

**Figure 5:**
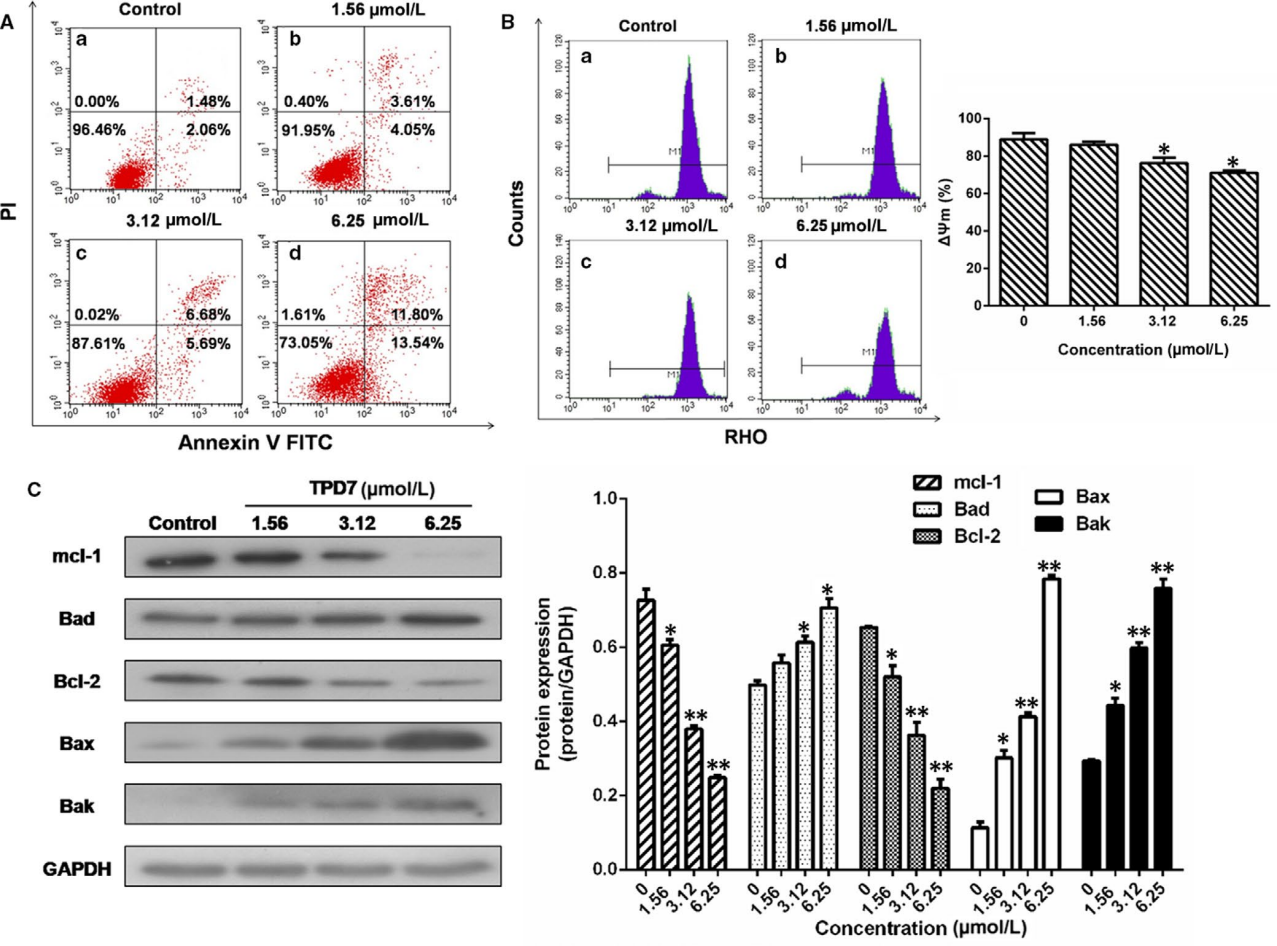
Effects of TPD7 treatment on H9 cell apoptosis. A, the proportion of apoptotic cells was determined by double staining with Annexin V‐FITC and propidium iodide in untreated control and H9 cells after treatment with TPD7 1.56, 3.12 and 6.25 μmol/L, respectively. The flow cytometry profile represents Annexin V‐FITC staining in x‐axis and PI in y‐axis. The number represents the percentages of cells to each of the four quadrants (viable cells for lower left quadrant, necrotic or dead cells in the higher left quadrant, early apoptotic cells in the lower right quadrant and late apoptotic cells in the higher right quadrant); B, effects of TPD7 on the mitochondrial membrane potential (Δψm). Δψm were assessed using flow cytometry following treatment of the H9 cells with TPD7 1.56, 3.12 or 6.25 μmol/L TPD7 for 48 h; C, effects of TPD7 on cell apoptosis‐related protein level. Data are presented as the mean ± standard deviation obtained from three independent experiments. *P < .05, **P < .01 and ***P < .001 vs. the control group

To examine whether apoptosis was initiated in H9 cells exposed to various concentrations of TPD7 for 48 hours, H9 cells were stained with Annexin V‐FITC and PI and explored by flow cytometry analysis. As shown in Figure 5A, after treatment with TPD7, the percentage of apoptotic cells increased to 7.66%, 12.37% and 25.34% respectively compared to control group (3.54%).

Based on the observation of TPD7‐induced cell apoptosis, the effect of TPD7 on mitochondrial transmembrane potential was examined to explore whether the mitochondrial pathway was responsible for TPD7‐induced apoptosis. The percentage of H9 cells exposed to TPD7 revealed a notable decrease in Rhodamine 123 fluorescence intensity from 88.97% in control to 86.02%, 76.31% and 63.06%, respectively (Figure 5B).

Since it has been observed that TPD7 could induce H9 cells apoptosis, we next investigated the level of apoptosis‐related proteins in Bcl‐2 family. The results showed that the amount of Bad, Bax and Bak was up‐regulated notably following treatment with TPD7, whereas Mcl‐1 and Bcl‐2 levels were decreased in a dose‐dependent manner (Figure 5C).

### 3.6 TPD7 regulates the downstream of IL‐2R signalling

**Figure 6:**
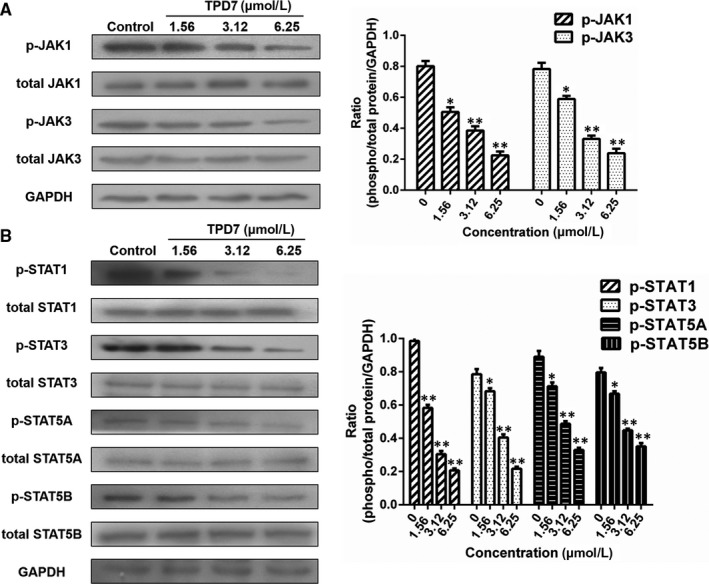
Effects of TPD7 on JAK/STAT signalling protein level. A, levels of JAK1, p‐JAK1, JAK3 and p‐JAK3 in H9 cells treated with TPD7 were examined by Western blot assay; B, levels of STAT1, p‐STAT1, STAT3, p‐STAT3, STAT5A, p‐STAT5A, STAT5B and p‐STAT5B in H9 cells treated with TPD7 were examined by Western blot assay. Data are presented as the mean ± standard deviation obtained from three independent experiments. *P < .05, **P < .01 and ***P < .001 vs. the control group

**Figure 7:**
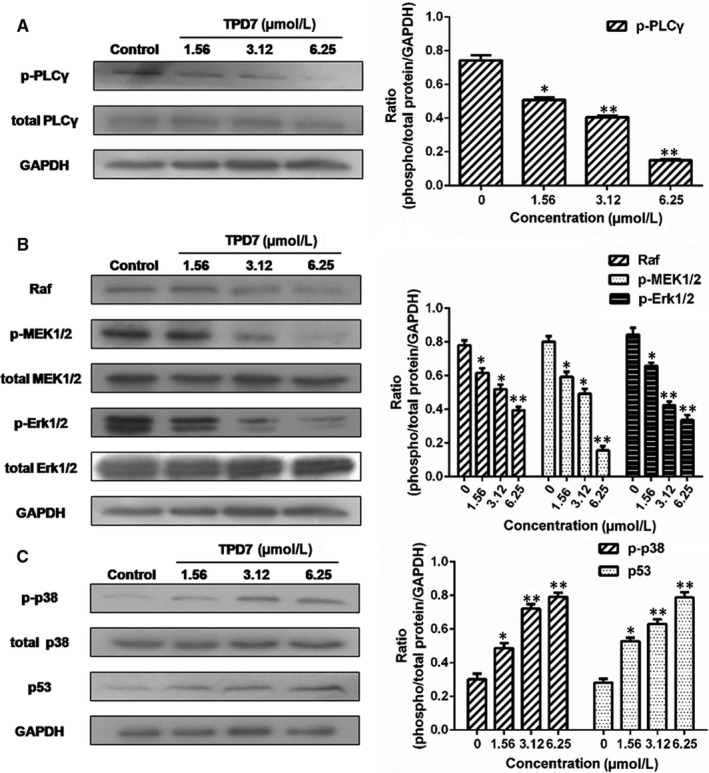
Effects of TPD7 on Raf/MAPK signalling protein level. A, levels of PLCγ and p‐PLCγ in H9 cells treated with TPD7 were examined by Western blot assay; B, levels of Raf, MEK1/2, p‐MEK1/2, Erk1/2 and p‐Erk1/2 in H9 cells treated with TPD7 were examined by Western blot assay; C, levels of p38, p‐p38 and p53 in H9 cells treated with TPD7 were examined by Western blot assay. Data are presented as the mean ± standard deviation obtained from three independent experiments. *P < .05, **P < .01 and ***P < .001 vs. the control group

**Figure 8:**
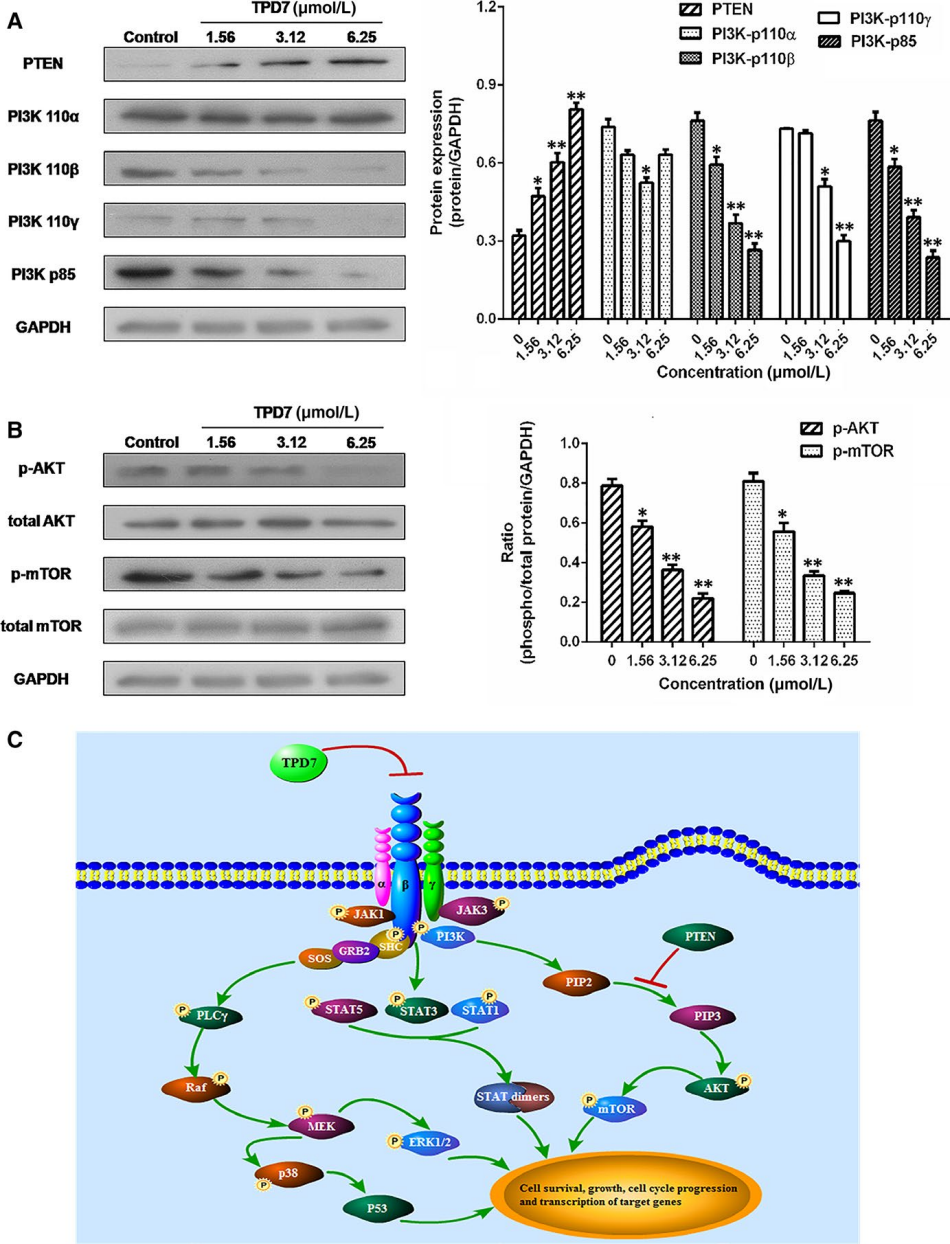
Effects of TPD7 on PI3K/AKT signalling protein level. A, levels of PTEN, PI3K‐p110α, PI3K‐p110β, PI3K‐p110γ and PI3K‐p85 in H9 cells treated with TPD7 were examined by Western blot assay; B, levels of AKT, p‐AKT, mTOR and p‐mTOR in H9 cells treated with TPD7 were examined by Western blot assay; C, potential demonstration of TPD7 inhibited the activation of IL‐2R signalling pathway of H9 cells. Data are presented as the mean ± standard deviation obtained from three independent experiments. *P < .05, **P < .01 and ***P < .001 vs. the control group

The major downstream molecules of IL‐2R signalling were tested as shown in Figures 6, 7, 8. The results showed that TPD7 effectively down‐regulated the phosphorylation of JAK/STAT signalling members including JAK1, JAK3, STAT1, STAT3, STAT5A and STAT5B (Figure 6). Meanwhile, treatment of TPD7 markedly suppressed the level of p‐PLC‐γ and Raf in a dose‐dependent manner and subsequently inhibited the phosphorylation of MEK1/2, leading to down‐regulation of p‐ERK1/2 and up‐regulation of p‐p38 and p53 (Figure 7). A significant decrease in the level of the main subunits of PI3K including 110β, 110γ and p85 and in the level of phosphorylation of AKT and mTOR were also observed in H9 cells treatment with TPD7 (Figure 8).

## 4 DISCUSSION

In the present study, we demonstrated a novel taspine derivative, TPD7, had a potent anti‐cancer activity against CTCL H9 cells via regulating the IL‐2R signalling pathway. We first sought the inhibitory effect of TPD7 in several different types of haematologic cancer cell lines including H9, HUT78, K562 and JURKAT cells, as well as the expression of IL‐2Rα, IL‐2Rβ and IL‐2Rγ in these cell lines. The results showed that H9 cells, whose expressions of IL‐2Rs were the highest level among these four cell lines, were most sensitive to TPD7 (Figure 1). Further characterization about the interaction between TPD7 and IL‐2R demonstrated that TPD7 interacted well with IL‐2R and meanwhile mRNA and protein level of IL‐2Rβ and IL‐2Rγ were significantly down‐regulated by TPD7 (Figures 2, 3). The obtained data were in consistent with our hypothesis that the potent inhibitory effect of TPD7 on H9 cell growth was due to the regulation of IL‐2R.

IL‐2, a pleiotropic cytokine, plays a critical role in the growth, proliferation and differentiation of T cells, which has also been verified to support the tumour cell growth of adult T cell leukaemia in vitro.^22^ Once binding to the receptors, IL‐2 activates multiple signalling pathways and thus plays its role effectively.^5^, ^6^ Our microarray analysis showed that TPD7 attenuated IL‐2Rβ and IL‐2Rγ genes in H9 cells and inhibited downstream pathway of IL‐2R including the major molecules of JAK/STAT, PI3K/AKT and MAPK pathway (Figure 3). The IL‐2Rα functions primarily by providing high affinity for IL‐2/IL‐2R system, while heterodimerization of IL‐2Rβ and IL‐2Rγ is involved in signal transduction through activation of JAKs, with JAK1 associating with IL‐2Rβ and JAK3 with IL‐2Rγ.^23^ Subsequently, different signalling pathways are activated depending on different key residues phosphorylated by activating IL‐2Rβ. Phosphorylation of specific tyrosine residues in the cytoplasmic domains of STAT5A, STAT5B, and, to a less extent, STAT3 and STAT1 recruited by IL‐2Rβ.^11^, ^24^ STAT5A and STAT5B are phosphorylated by IL‐2Rβ activation and thereafter dimerized and translocated to the nucleus, where they can take effect on T cell growth by binding to target genes.^24^ As a promising target for cancer therapy, STAT3 is not only crucial for transducing signals through regulating the expression of a wide range of genes, but also has a crucial role in stromal cells, including immune cells through recruiting them to the tumour microenvironment, thereby contributing to tumour progression.^25^ Although STAT1 is generally regarded as a tumour suppressor, there is increasing evidence that it could promote tumour cell growth and invasiveness, suppress tumour immune surveillance and induce therapy resistance against irradiation and chemotherapy.^26^ In our study, the phosphorylation level of JAK1, JAK3, STAT1, STAT3, STAT5A and STAT5B is all decreased dramatically, which indicated TPD7 inhibited JAK/STAT signalling pathway via suppressing the expression of IL‐2R (Figure 6). Furthermore, the PI3K/AKT/mTOR signalling pathway can also be activated by IL‐2R, which is relevant to tumour growth, cell survival, cell proliferation, cell cycle progression and apoptosis.^27^ In addition, it has been revealed that the essential tumour suppressor gene PTEN encodes a phosphatase protein against the PI3K/AKT anti‐apoptotic pathway.^28^ Our results showed that PTEN was up‐regulated and the amount of several major subunits of PI3K including 110β, 110γ and p85 were significantly reduced following TPD7 treatment in H9 cells, as well as the phosphorylation level of downstream signal molecules (AKT and mTOR) (Figure 8). Many studies have identified that the Raf/MAPK pathway involved in tumour development and progression.^29^ Importantly, activation of IL‐2Rβ is also capable of activating PLCγ/Raf/MAPK signalling pathway, which plays a pivotal role in regulating cell proliferation and survival.^30^, ^31^ ERK(ERK1/2) and p38 MAPK, two of the major classical MAPK pathway, have been proposed as targets in cancer therapy as a result of abnormal activation.^32^ Results showed that TPD7 significantly decreased the level of p‐PLCγ, Raf, p‐MEK1/2 and p‐ERK1/2, while increased the level of p‐p38 and p53 (Figure 7). These data confirmed that TPD7 regulated IL‐2R signalling and related downstream signal transductions in order to inhibit the H9 cell growth.

Since the downstream cascade of PI3K/AKT pathway and MAPK pathway are involved in cell cycle progression and cell apoptosis, the effect of TPD7 on cell viability could be mediated by changes in proliferation, apoptosis or both. Our expression profiles indicated that cell apoptotic process and cell cycle machinery were involved in the gene differences in TPD7‐treated H9 cells (Figure 3). The cell cycle is an intricate controlled process implicated in cell growth and division and is susceptible to modulation in cancer cells owing to the rapid proliferation rate. We next focused on the effect of TPD7 treatment on cell cycle progression and the results showed that TPD7 mainly blocked the cell cycle progression at G2/M phase and partially at G1 phase (Figure 4). Previously, cyclin‐dependent kinase (CDK) and cyclins function as dynamic regulators of cell cycle progression.^33^ CyclinB1 was associated with the G2/M checkpoint control and could form a complex by binding with CDC2, which takes an important part in G2/M phase transition.^34^ And, it was well recognized that CDC2, cyclinD1 and cyclinE are critical molecules in the regulation of cells from G1 phase into S phase.^35^ Consistent with these, we observed a decrease in CDC2, cyclinB1, cyclinD1 and cyclinE level in H9 cells treated with TPD7 (Figure 4).

It is well‐known that apoptosis, as one of the most important signal of cell death, is typically displayed unbalanced regulation in human cancers and evasion of apoptosis is an universal hallmark of cancer.^36^ Besides, the intrinsic mitochondrial pathway is one of the major pathways to induce programmed cell death. Flow cytometry results revealed that TPD7 treatment induced a pronounced apoptosis and the loss of Δψm in H9 cells (Figure 5). We therefore sought to determine the underlying molecular mechanisms of TPD7‐induced apoptosis in the mitochondrial pathway. Bcl‐2 family proteins play a pivotal role in regulating cell apoptosis due to their ability to form membrane channels in mitochondria, which can be divided into two subfamilies: pro‐apoptotic members (Bcl‐2, Mcl‐1, etc) and anti‐apoptotic members (Bax, Bak, Bad, etc).^37^ Localized to the outer membrane of mitochondria, Bcl‐2 has a significant impact on mitochondrial integrity. Our results verified that TPD7 significantly decreased the levels of the anti‐apoptotic Bcl‐2 and Mcl‐1 proteins and increased the levels of the pro‐apoptotic Bax, Bak and Bad proteins in H9 cells (Figure 5). Thus, the effect of TPD7 in inhibiting H9 cell viability appears to be mediated by induction of cell cycle arrest and increased cell apoptosis through the mitochondrial pathway.

Taken together, we concluded that IL‐2Rs are highly expressed in H9 and HUT78 cell lines and can be an effective target for CTCL targeted therapy. Moreover, TPD7 exerts its potent inhibitory effect on H9 cell growth by impairing the expression of IL‐2Rβ and IL‐2Rγ and thus modulating JAK/STAT, PI3K/AKT/mTOR and PLCγ/Raf/MAPK signalling, leading to cell cycle arrest at G2/M phase by regulating CDK/Cyclins proteins and induction of cell apoptosis by regulating Bcl‐2 mitochondrial pathway (Figure 8). Based on these results, TPD7 may be a potential candidate for cutaneous T cell lymphoma chemotherapy.

## CONFLICTS OF INTEREST

The authors declare that there are no conflicts of interest to disclose.

## AUTHOR CONTRIBUTIONS

Weina Ma designed the study, interpreted the data and edited the manuscript; Man Zhu wrote the manuscript, designed the study, analysed and interpreted the data; Liu Yang and Xianpeng Shi designed the study, interpreted the data and edited the manuscript; Dongdong Zhang and Yanmin Zhang helped in the study design and edited the manuscript; Zhengyan Gong and Runze Yu reviewed the manuscript and helped in the study design. All authors approved the final version of the manuscript.

## Data Availability

The authors declare that all the data supporting the findings of this study are available within the article.
